# High mammographic breast density predicts locoregional recurrence after modified radical mastectomy for invasive breast cancer: a case-control study

**DOI:** 10.1186/s13058-016-0784-3

**Published:** 2016-12-01

**Authors:** Yu-Sen Huang, Jenny Ling-Yu Chen, Chiun-Sheng Huang, Sung-Hsin Kuo, Fu-Shan Jaw, Yao-Hui Tseng, Wei-Chun Ko, Yeun-Chung Chang

**Affiliations:** 1 Institute of Biomedical Engineering, College of Medicine and College of Engineering, National Taiwan University, Taipei, Taiwan; 2 Department of Medical Imaging, National Taiwan University Hospital and National Taiwan University College of Medicine, No. 7, Chung-Shan S. Rd., Taipei, 100 Taiwan; 3 Department of Medical Imaging, National Taiwan University Hospital Yun-Lin Branch, Yun-Lin, Taiwan; 4 Department of Oncology, National Taiwan University Hospital and National Taiwan University College of Medicine, Taipei, Taiwan; 5 Department of Radiation Oncology, National Taiwan University Hospital Hsin-Chu Branch, Hsin-Chu, Taiwan; 6 Department of Surgery, National Taiwan University Hospital and National Taiwan University College of Medicine, Taipei, Taiwan

**Keywords:** Mammographic breast density, Breast cancer, Modified radical mastectomy, Loco-regional recurrence, Distant metastasis, Case-control study

## Abstract

**Background:**

We aimed to evaluate the influence of mammographic breast density at diagnosis on the risk of cancer recurrence and survival outcomes in patients with invasive breast cancer after modified radical mastectomy.

**Methods:**

This case-control study included 121 case-control pairs of women diagnosed with invasive breast cancer between 2004 and 2009, and who had undergone modified radical mastectomy and had mammographic breast density measured before or at diagnosis. Women with known locoregional recurrence or distant metastasis were matched by pathological disease stage, age, and year of diagnosis to women without recurrence. Locoregional recurrence was defined as recurrence in the ipsilateral chest wall, or axillary, internal mammary, or supraclavicular nodes. The median follow-up duration was 84.0 months for case patients and 92.9 months for control patients.

**Results:**

Patients with heterogeneously dense (50–75% density) and extremely dense (>75% density) breasts had an increased risk of locoregional recurrence (hazard ratios 3.1 and 5.7, 95% confidence intervals 1.1–9.8 and 1.2–34.9, *p* = 0.043 and 0.048, respectively) than did women with less dense breasts. Positive margins after surgery also increased the risk of locoregional recurrence (hazard ratio 3.3, 95% confidence interval 1.3–8.3, *p* = 0.010). Multivariate analysis that included dense breasts (>50% density), positive margin, no adjuvant radiotherapy, and no adjuvant chemotherapy revealed that dense breasts were significant factors for predicting locoregional recurrence risk (hazard ratio 3.6, 95% confidence interval 1.2–11.1, *p* = 0.025).

**Conclusions:**

Our results demonstrate that dense breast tissue (>50% density) increased the risk of locoregional recurrence after modified radical mastectomy in patients with invasive breast cancer. Additional prospective studies are necessary to validate these findings.

**Trial registration:**

The study is retrospectively registered with ClinicalTrials.gov, number NCT02771665, on May 11, 2016.

## Background

Breast cancer remains the most commonly diagnosed cancer and the leading cause of cancer-related death in women worldwide [1]. Mammographic breast density (MBD) refers to the tissue composition of the breast. The epithelium and fibrous tissue are radiodense and appear white on a mammogram, whereas the fatty tissue is radiolucent and appears black. MBD is calculated by dividing the dense area by the total breast area. Larger amounts of fibroglandular tissue in relation to fatty tissue will lead to higher MBD values. High MBD has been associated with increased risk of breast cancer [2, 3] and local recurrence of invasive breast cancer after breast-conserving surgery [4–6]. Several possible mechanisms by which density could affect prognosis have been investigated. MBD has consistently been associated with breast stromal composition, which is involved in tumor progression [7–10].

Modified radical mastectomy (MRM) has been the primary treatment method for local breast cancer [11, 12]. According to published data, nearly one tenth of mastectomized patients are at risk of locoregional recurrence (LRR), and a quarter of patients are at risk of developing distant metastases during follow up [13]. We hypothesized that a high MBD and a microenvironment rich in extracellular matrix (ECM) might promote cancers that are more aggressive. This study therefore aimed to evaluate the influence of high MBD on the risk of recurrence in patients with invasive breast cancer after MRM.

## Methods

This study was approved by the Institutional Review Board at National Taiwan University Hospital (approval number 201401067RIND). The patients’ medical data were anonymized prior to access and analysis. The Institutional Review Board at NTUH waived the need for written informed consent from the study subjects because all potentially patient-identifying information was removed before data analysis.

**Fig. 1:**
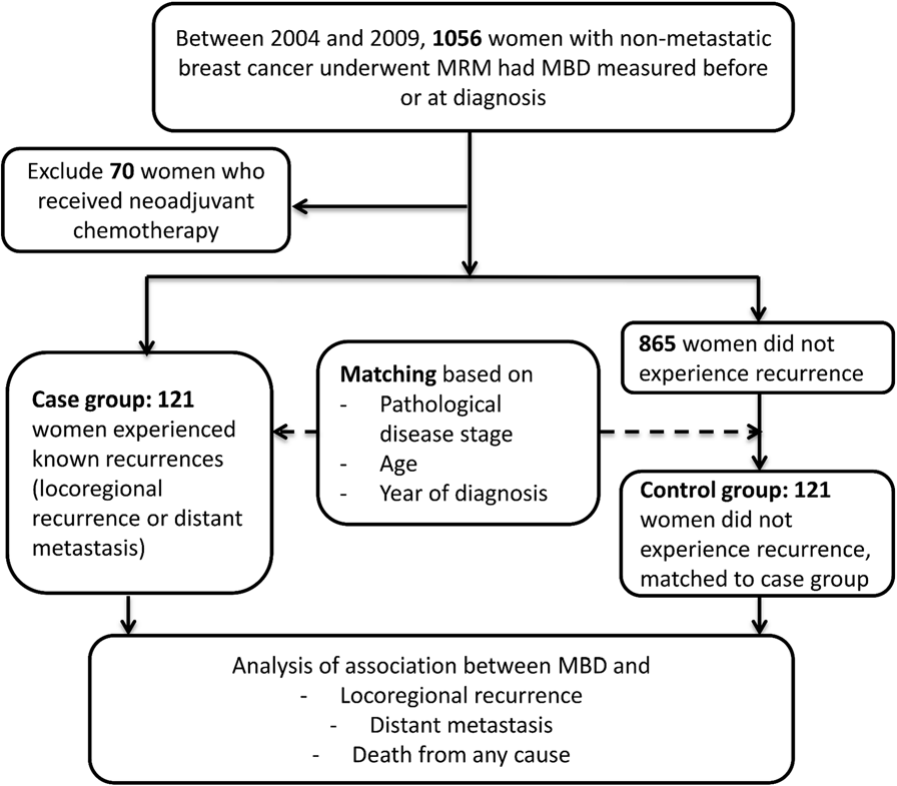
Patient flowchart. Between January 2004 and December 2009, a total of 1056 patients received modified radical mastectomy (*MRM*) and had pre-operative mammographic breast density (*MBD*) measured at our institution. Seventy women who received neoadjuvant chemotherapy were excluded. Recurrences were categorized as locoregional (ipsilateral chest wall or axillary, internal mammary, or supraclavicular nodes) or distant metastasis. Each patient with known locoregional recurrence or distant metastasis (n = 121, the case group) were matched to at least one patient without recurrence (the control group) by pathological disease stage, age, and year of diagnosis

Between January 2004 and December 2009, a total of 4089 women were diagnosed and treated with invasive, non-metastatic breast cancer at our institution, 2011 of whom underwent definitive MRM. Approximately 20% of the MRM cases were diagnosed and staged at regional hospitals and later referred to our institution (the official tertiary referral hospital) for definitive surgery; consequently, the pre-surgery mammograms were not registered in our image database. Moreover, the image database in our institution underwent a major upgrade in 2005, which resulted in a partial data loss. Therefore, at the time of analysis, there were 1056 pre-surgery mammograms in our image database for MBD measurement. Among these 1056 patients who received MRM and had pre-operative MBD measured, 38% had stage I disease, 34% had stage II disease, and 28% had stage III disease. Seventy were excluded from the analysis because the patients had received neoadjuvant chemotherapy. Recurrence was categorized as locoregional (ipsilateral chest wall or axillary, internal mammary, or supraclavicular nodes) or distant metastasis (DM). A total of 121 patients experienced either LRR or DM. The patient flow chart is shown in Fig. 1.

Case control matching was based on pathological disease stage as defined by the American Joint Committee on Cancer, patient age, and year of diagnosis. Each patient with recurrence was matched with at least one control. Control patients had breast cancer but no recurrence. Matching patients according to exact disease stage, age, and year of diagnosis was possible in only a few instances; therefore, the age criterion was relaxed to allow age differences within 3 years. Subject demographic information, self-reported height and weight, breast cancer risk factors, tumor characteristics (e.g., tumor size, axillary node status, and surgery margin), and adjuvant treatment information were obtained from the Cancer Registry Medical Information Management Office at our institution. Ultimately, 121 women with LRR or DM were matched to 121 women without recurrence. The patient flow chart is shown in Fig 1.

Tumors scored as human epidermal growth factor receptor 2 (Her2) 3+ or as 2+ with *HER2* amplification (determined via fluorescence *in situ* hybridization, ratio ≥2) were considered Her2-positive. Owing to our country’s national health insurance policy, our national health insurance program completely covers hormone-receptor-positive patients, and selectively covers adjuvant trastuzumab therapy in patients with Her2-positive disease and positive nodal status.

Follow-up analysis was conducted using a comprehensive protocol and the relevant data available on 10 September 2016. All patients were followed up every 3 months in the first 2 years, every 4 months in the third and fourth years, and then every 6 months until recurrence or death. The follow-up visits included physical examinations, determination of tumor marker expression, and imaging studies if required. Recurrence was defined only by the site of the first relapse (i.e., locoregional or distant). The median length of follow up in case patients was 84.0 months (range 23.9–148.4 months) and in control patients it was 92.9 months (range 18.2–150.7 months). All but two patients were followed up for at least 36 months [14]; the exceptions were one control patient with a follow-up period of 23.9 months and one case patient with a follow-up period of 18.2 months.

### MBD measurements

MBD was defined on a mammogram of the contralateral breast (i.e., the breast not affected by cancer) taken any time during the year preceding the MRM. MBDs in all patients were measured using a previously described, validated, computer-based interactive threshold method [15]. A dedicated mammographic unit (GE, Senographe DMR, Buc, Cedex, France) with an 18 × 24-cm Min-R M or Min-R 2000 screen/film system (Eastman Kodak, Rochester, NY, USA) was used for all patients [16]. All mammographic images were read by radiologists with at least 5 years of experience in breast imaging. We used the public ImageJ software to establish the semi-automated process for evaluating MBD on digital mammograms [17]. The mediolateral oblique was the preferred mammographic projection for MBD evaluation.

**Fig. 2:**
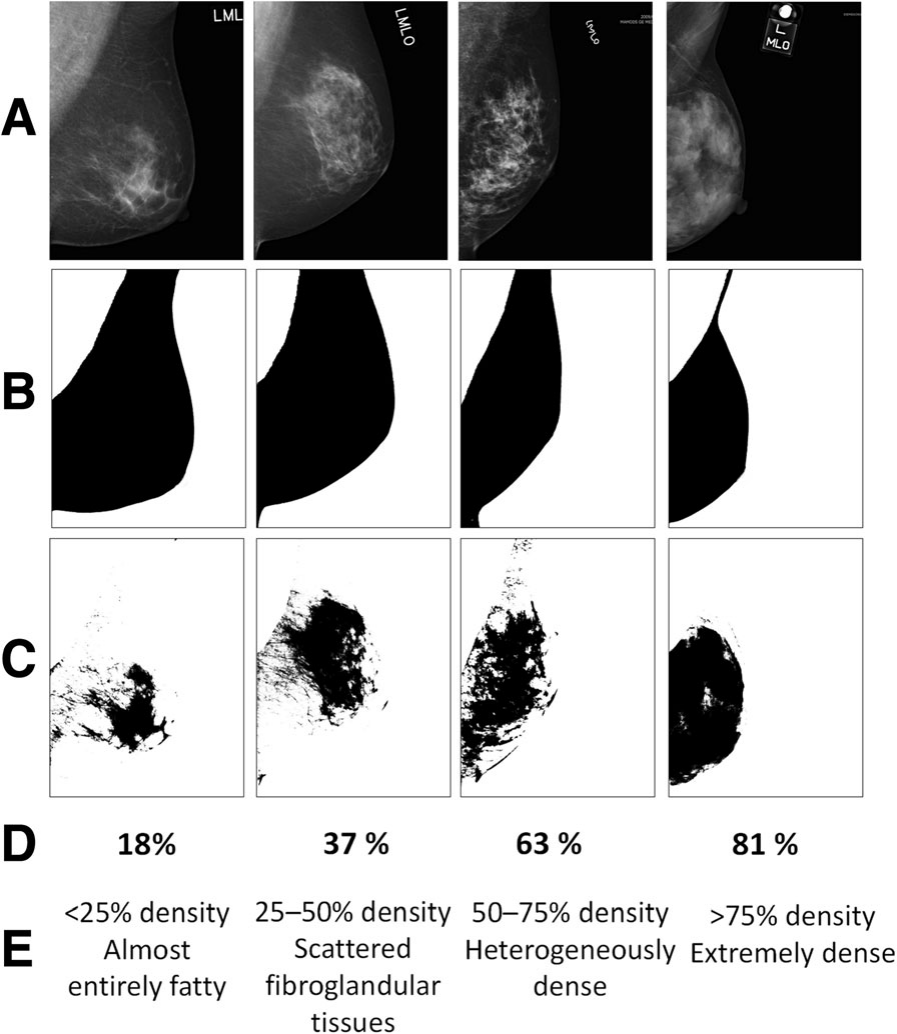
Mammographic breast density measurement using the computer-assisted method. **a** Left mediolateral oblique projection mammograms were used to assess women with right-sided breast cancer. **b** A threshold was selected by the operator to isolate the breast from the surrounding background. **c** A second threshold was set to identify areas of density. **d** The mammographic breast density (MBD) percentage was determined by measuring the total area of the breast and the number of pixels outlined in the dense regions using the computer software. **e** The estimated MBD was classified according to the American College of Radiology (ACR) lexicon definition before 2013. Specifically, ACR 1 indicates <25% density and that “the breast is almost entirely fat.” ACR 2 indicates 25–50% density and that “there are scattered fibroglandular tissues.” ACR 3 indicates 50–75% density and that “the breast is heterogeneously dense.” ACR 4 indicates >75% density and that “the breast is extremely dense”

In brief, each digitized mammogram was displayed on a computer screen, and a threshold was selected by the operator to isolate the breast from the surrounding background (Fig. 2). A second threshold was set to identify areas of density. The computer-assisted method divided the mammographic image according to a gray-value distribution, with darker regions indicating fatty tissue and lighter regions representing dense tissue. The MBD percentage was determined by measuring the total breast area and the number of pixels outlined in the dense regions using the computer software. The breast composition of each patient, as revealed by the mammograms, was estimated according to the American College of Radiology (ACR) lexicon definition before 2013. Specifically, ACR 1 indicates <25% density and that “the breast is almost entirely fat.” ACR 2 indicates 25–50% density and that “there are scattered fibroglandular tissues.” ACR 3 indicates 50–75% density and that “the breast is heterogeneously dense.” ACR 4 indicates >75% density and that “the breast is extremely dense.” There was strong correlation between the externally validated results using the ACR lexicon definition interpreted by the radiologists and the computer-assisted method (*R* ^2^ = 0.906, *p* <0.001).

### Statistical analysis

Statistical assistance was provided by the Clinical Trial Bioinformatics and Statistical Center, Training Center, and Pharmacogenomics Laboratory, founded by the National Research Program for Biopharmaceuticals at the Ministry of Science and Technology (MOST 103-2325-B-002-033) in our country, and the Department of Medical Research at our institution. The Statistical Package for Social Sciences for Windows, version 17.0 (SPSS Inc., Chicago, IL, USA), was used for statistical analysis. Survival data were confirmed with the Cancer Registry Medical Information Management Office at our institution. All events were calculated from the date of MRM completion.

Descriptive characteristics were compared using the chi-squared test for categorical variables and the paired Student’s *t* test for continuous variables. We assessed the association between MBD and recurrence or death from any cause using a conditional logistic regression model. Conditional logistic regression analyses with stratified Cox models were used to evaluate the potential risk factors for patients with LRR. Prognostic variables found to be significant at *p* <0.20 on univariate analysis were included in a multivariate model and then removed, one by one, by eliminating the variable with the greatest *p* value and retesting the remaining variables using the Cox proportional hazards regression model. Overall, a *p* value ≤0.05 was considered statistically significant.

## Results

The patient flow chart is shown in Fig 1. We recruited 121 case-control pairs for our analysis, which included 43 case patients who experienced LRR and 78 who experienced DM. In the 43 LRR case patients, there were 18 chest wall, 17 axillary node, one internal mammary node, three supraclavicular node, one synchronous chest wall and internal mammary node, one synchronous internal mammary and supraclavicular node, and two synchronous chest wall and axillary node recurrences.

Table 1 lists the clinical characteristics of the case patients and control subjects, who were matched according to pathological disease stage, age, and year of diagnosis. The median age was 51.6 years (range 27–94 years), and the mean body mass index (BMI) was 23.6 kg/m^2^ (range 15.4–38.1 kg/m^2^). Among the case and control patients, 38.0% of the patients had stage III disease. There were no significant differences in tumor size or number of positive axillary nodes between case patients and control patients. Triple-negative breast cancers were observed in 21.5% of case patients and 17.4% of control patients (*p* = 0.417). Dense breasts (MBD >50%) were observed more frequently in the case patients than in the control patients, although this difference was not statistically significant (43.0% vs. 32.2%, *p* = 0.092).

**Table 1:** Characteristics between patients who did or did not experience recurrences

	Control group		Case group		
	*n* = 121	Percentage	*n* = 121	Percentage	*P* value
Age (years)					
mean (range)	51.4 (28–89)		51.6 (27–94)		0.738*
<40	15	12.4	19	15.7	0.725^†^
40–59	87	71.9	82	67.8	
>60	19	15.7	20	16.5	
AJCC stage (pathological)					
I	24	19.8	24	19.8	1.000^†^
IIA	33	27.3	33	27.3	
IIB	18	14.9	18	14.9	
IIIA	20	16.5	20	16.5	
IIIB	2	1.7	2	1.7	
IIIC	24	19.8	24	19.8	
Tumor size (mm)					
0–10	19	15.7	19	15.7	0.911^†^
10 − 19	19	15.7	22	18.2	
20 − 50	68	56.2	63	52.1	
>50	15	12.4	17	14.0	
Positive axillary nodes					
None	47	38.8	49	40.5	0.956^†^
1–3	32	26.4	32	26.4	
>4	42	34.7	40	33.1	
Margin					
Negative	112	92.6	106	87.6	0.197
Positive	9	7.4	15	12.4	
Estrogen/progesterone receptor status					
Negative	46	38.0	44	36.4	0.790^†^
Positive	75	62.0	77	63.6	
Her2 status^a^					
Negative	92	76.0	88	72.7	0.556
Positive	29	24.0	33	27.3	
Triple-negative cancer					
Negative	100	82.6	95	78.5	0.417
Positive	21	17.4	26	21.5	
MBD quartiles					
1 (0–25%)	29	24.0	10	8.3	0.023^†^
2 (25–50%)	53	43.8	59	48.8	
3 (50–75%)	32	26.4	41	33.9	
4 (75–100%)	7	5.8	11	9.1	
Adjuvant chemotherapy					
No	26	21.5	21	17.4	0.417^†^
Yes	95	78.5	100	82.6	
Adjuvant hormone therapy					
No	47	38.8	50	41.3	0.694^†^
Yes	74	61.2	71	58.7	
Adjuvant target therapy					
No	100	82.6	102	84.3	0.729^†^
Yes	21	17.4	19	15.7	
Adjuvant radiotherapy					
No	59	48.8	64	52.9	0.520^†^
Yes	62	51.2	57	47.1	
BMI (kg/m^2^)					
mean (range)	24.0 (16.2–35.4)		23.1 (15.4–38.1)		0.272*
Normal (<25)	85	70.2	80	64.5	0.627^†^
Overweight (25–30)	28	23.1	33	27.3	
Obese (>30)	8	6.6	10	8.3	

^a^ Human epidermal growth factor receptor 2 (Her2) status indicated all Her2 (3+)-positive tumors and Her2 (2+)- positive tumors with *Her2* amplification investigated by fluorescence in situ hybridization; ratio ≥ 2. *AJCC* American Joint Committee on Cancer, *MBD* mammographic breast density, *BMI* body mass index. *Significance tested using paired Student’s *t* test. ^†^Significance tested using Pearson’s chi-square test

At the time of analysis, 53 case patients and eight control patients had died. The median length of follow up was 84.0 months (range 23.9–148.4 months) in case patients and 92.9 months (range 18.2–150.7 months) in control patients. Statistically significant differences in the likelihood of LRR, DM, or death from any cause were observed between women with MBD values <50% and those with MBD values between 50% and 75% or MBD >75%. As shown in Table 2, patients with heterogeneously dense (50–75% density) and extremely dense (>75% density) breasts had an increased risk of LRR (hazard ratios 3.1 and 5.7, 95% confidence intervals 1.1–9.8 and 1.2–34.9, *p* = 0.043 and 0.048, respectively). We found no association between MBD and the risk of distant metastasis or death from any cause.

**Table 2:** Hazard ratios of cancer recurrence and survival outcome based on MBD quartiles (*n* = 121 case-control pairs)

	Locoregional recurrence			Distance metastasis			Death		
	HR	95% CI	*P* value*	HR	95% CI	*P* value*	HR	95% CI	*P* value*
MBD quartiles									
1, 2 (0–50%)	1.0			1.0			1.0		
3 (50–75%)	3.1	1.1–9.8	0.043^†^	5.7	0.0–21.9	0.492	3.2	0.9–10.1	0.062
4 (75–100%)	5.7	1.2–34.9	0.048^†^	10.4	0.8–37.2	0.311	2.2	0.3–13.7	0.409

*MBD* mammographic breast density, *HR* hazard ratio, *CI* confidence interval. *Significance tested using conditional logistic regression analyses by stratified Cox model. ^†^ *P value* <0.05 was considered statistically significant

The results of the univariate analysis of potential factors affecting LRR in the 121 case-control pairs are shown in Table 3. There was no significant correlation between LRR and tumor size, number of positive axillary nodes, type of adjuvant therapy, triple-negative cancer, or BMI. However, positive margins after surgery increased the risk of LRR (hazard ratio 3.3, 95% confidence interval 1.3–8.3, *p* = 0.010). Although not significant, no adjuvant radiotherapy (*p* = 0.120) or no adjuvant chemotherapy (*p* = 0.166) also increased the risk of LRR.

**Table 3:** Univariate analysis of risk factors associated with locoregional recurrence (*n* = 121 case-control pairs)

	Control group	Case group (all patients)	Case group (only patients with LRR)			
	*n* = 121	*n* = 121	*n* = 43	HR	95% CI	*P* value*
MBD quartiles						
1, 2 (0– 50%)	82	69	26	1.0		
3 (50– 75%)	32	41	13	3.1	1.1–9.8	0.043^†^
4 (75– 100%)	7	11	4	5.7	1.2–34.9	0.048^†^
Tumor size (mm)						
0–10	19	19	8	1.0		
10 − 19	19	22	12	2.5	0.6–10.1	0.187
20 − 50	68	63	15	1.8	0.2–3.8	0.806
>50	15	17	8	4.1	0.3–57.5	0.291
Positive axillary nodes						
None	47	49	15	1.0		
1–3	32	32	12	0.6	0.1–2.5	0.477
>4	42	40	16	0.8	0.1–6.4	0.731
Margin						
Negative	112	106	37	1.0		
Positive	9	15	6	3.3	1.3–8.3	0.010^†^
Adjuvant chemotherapy						
Yes	95	100	32	1.0		
No	26	21	11	2.0	0.7–5.3	0.166
Adjuvant hormone therapy						
Yes	74	71	22	1.0		
No	47	50	21	2.0	0.2–22.1	0.571
Adjuvant target therapy						
Yes	21	19	6	1.0		
No	100	102	37	6.5	0.0–56.5	0.471
Adjuvant radiotherapy						
Yes	62	57	20	1.0		
No	59	64	23	1.6	0.6–3.9	0.120
Triple-negative cancer						
Negative	100	95	34	1.0		
Positive	21	26	9	2.0	0.5–8.0	0.327
BMI (kg/m^2^)						
Normal (<25)	85	80	26	1.0		
Overweight (25–30)	28	33	13	1.2	0.6–2.4	0.708
Obese (>30)	8	10	4	1.1	0.3–3.8	0.931

*LRR* locoregional recurrence, *MBD* mammographic breast density, *BMI* body mass index, *HR* hazard ratio, *CI* confidence interval. *Significance tested using conditional logistic regression analyses by stratified Cox model. ^†^ *P* value <0.05 was considered statistically significant

To determine which factors were independently associated with LRR, we performed stepwise elimination logistic regression analysis using the Cox proportional hazards regression model and the variables with *p* values <0.20 in the univariate analysis (no adjuvant radiotherapy, no adjuvant chemotherapy, positive margins, and MBD >50%). On multivariate analysis (Table 4), dense breasts were a significant factor for predicting risk of LRR (hazard ratio 3.6, 95% confidence interval 1.2–11.1, *p* = *0.025*).

**Table 4:** Multivariate analysis of risk factors associated with locoregional recurrence (*n* = 121 case-control pairs)

	HR	95% CI	*P* value*
Dense breasts (MBD >50%)	3.6	1.2-11.1	0.025^†^
Positive margin	2.9	0.9-9.3	0.077
Adjuvant radiotherapy	2.2	0.9-5.9	0.100
Adjuvant chemotherapy	2.3	0.8-6.7	0.135

*MBD* mammographic breast density, *HR* hazard ratio, *CI* confidence interval. *Significance tested using proportional hazards regression analyses by stratified Cox model. ^†^ *P* value <0.05 with statistical significance. ^†^ *P* value <0.05 was considered statistically significant

## Discussion

The present study shows that high MBD is a significant independent predictor of LRR after MRM in patients with invasive breast cancer. To our knowledge, it is one of the few studies published to date to have evaluated breast density as a predictor of breast cancer recurrence, with generally consistent results [4–6, 18]. Park et al. [5] conducted a case-control study of 136 patients with invasive breast cancer who underwent breast-conserving surgery and radiotherapy, and found that those with the highest breast density (>75%) had a greater than fourfold incidence of local, but not distant, disease recurrence compared to those with low breast density (<25%). Cil et al. [6] conducted a retrospective cohort study of 355 patients who underwent breast-conserving surgery for invasive breast cancer and demonstrated fivefold higher risk of local disease recurrence in patients with high (>50%) versus low <25%) mammographic density. Eriksson et al. [4] conducted a case-only study of 1774 patients who underwent breast-conserving surgery or mastectomy for invasive breast cancer and found nearly twofold higher risk of locoregional recurrence in women with MBD >25% versus <25%, and MBD was not associated with either distant recurrence or survival.

To reduce the possibility of recurrence being overlooked, our institution has a comprehensive follow-up protocol. When all women with invasive breast cancer treated by MRM at our institution were analyzed, the LRR rate was 4.1% (43 of 1056 patients), and among these patients, 38% had stage I disease, 34% had stage II disease, and 28% had stage III disease. Among the 121 case patients, nearly 60% had stage I/II disease and nearly 40% had stage III disease. The lack of correspondence between the distribution of disease stage in the overall cohort (1056 patients who received MRM and had pre-operative MBD measured) and case cohort (121 patients with 43 LRRs and 78 DMs) is reasonable, as the case patients were not randomly selected from the overall cohort. Half of the case patients with an LRR had received post-mastectomy radiotherapy, and our LRR rate of 4.1% is consistent with the LRR rates (1.6–8.1%) obtained in a study by the Early Breast Cancer Trialists’ Collaborative Group [19], in which patients received mastectomy and adjuvant radiotherapy.

The percentage of patients with positive nodes was not different between the control cohort and case cohort (*p* = 0.956, Table 1). We did not observe a significant difference in the hazard ratio among patients with positive nodes, which may be due to the case-control study design, as we matched the case patients to the control patients with the same disease stage to eliminate this confounding factor, as there was strong correlation between the tumor, node, metastatis (TNM) staging system and locoregional recurrence.

We found no significant impact of breast density on distant recurrence or death. Risk factors for LRR are not the same as those for death because there are reasonable salvage treatments, which may partly explain why high breast density is not significantly correlated with death. Gierach et al. [20] reported that breast imaging-reporting and data system (BIRADS) density categories are not related to the risk of death, which is consistent with our study finding. Interestingly, they pointed out that patients with breast cancer with a low breast density, and who are obese (BMI >30 kg/m^2^) have increased risk of death; this is probably due to increased BMI, larger adipocyte size, and breast microenvironments that provide a stimulus for tumor growth, which was not seen in our study. More comprehensive information, including recurrence patterns, health comorbidities, and cause of death, is needed to identify the impact of obesity in patients with low breast density. There were only a few obese patients (one tenth) in our cohort, whereas one fourth of patients were obese in the Gierach study; hence, obesity is less likely to be positively correlated with death in our cohort due to the small number of patients. Results from Western society may not be applicable to our populations because of the unique etiology, molecular subtype, invasion pattern, recurrence, or prognosis among our patients with breast cancer [21].

Our results indicate that high MBD values independently confer greater LRR risk upon women with breast cancer, but not distant metastasis. MBD reflects the breast composition, and higher values indicate larger proportions of fibroglandular elements and extracellular matrix (ECM) relative to adipose stroma [8, 9]. An abnormal microenvironment and dysregulated cell–ECM matrix signaling have been associated with breast cancer invasion, metastasis, and therapeutic resistance [13, 22]. From a biological perspective, high MBD is suggestive of enhanced migratory, invasive, and metastatic behavior resulting from interactions of the ECM with the surrounding tumor and blood vessel walls [7, 10]. Increased ECM signaling, and particularly β1-integrin expression, has also been associated with reduced survival in patients with invasive breast cancer [23]. One hypothesis for LRR after mastectomy in patients with high MBD is that MBD can increase the risk of self-seeding, a process in which disseminated tumor cells return and colonize the primary tumor site [24]. In contrast to metastasis, which requires the ability to enter, survive, and colonize a new site, self-seeding needs little or no adaptation, as the circulating tumor cell returns to a familiar environment [25]. This would explain the phenomenon of post-mastectomy LRR.

There has been considerable interest in modifiable risk factors that may prevent recurrent disease and in the potential use of breast density as an intermediate risk biomarker. We performed multivariate regression analysis to account for possible collinearities, including adjuvant radiotherapy or chemotherapy [26], positive margin, and MBD >50%, to determine if MBD independently confers increased risk of LRR. Our data support the hypothesis that MBD may be used as a practical prognostic factor. However, it remains unclear whether MBD can be used to discriminate greater LRR risk in all breast cancer patients. This is an issue to be addressed in future larger studies.

Our study was limited by its case-control design. In addition, half of the patients in the initial cohort had to be excluded from the analysis because their pre-surgery mammograms were not available. This exclusion is a potential source of bias if it was not random. However, analysis of the comparison statistics confirmed that the excluded patients did not differ from those included in the analysis in important factors such as disease stage, age, and year of diagnosis.

## Conclusions

In the present study, we found that high MBD (>50% density) is a significant independent predictor of LRR after MRM for invasive breast cancer. The results of this study might influence patient management and may be helpful to physicians when making clinical decisions. Additional prospective studies are necessary to validate these findings.

## Abbreviations

ACR: American College of Radiologists
BMI: Body mass index
DM: Distant metastasis
ECM: Extracellular matrix
Her2: Human epidermal growth factor receptor 2
LRR: Locoregional recurrence
MBD: Mammographic breast density
MRM: Modified radical mastectomy

## References

[1] Jemal A, Center MM, DeSantis C, Ward EM (2010). Global patterns of cancer incidence and mortality rates and trends. Cancer Epidemiol Biomarkers Prev.

[2] Boyd NF, Guo H, Martin LJ, Sun L, Stone J, Fishell E, Jong RA, Hislop G, Chiarelli A, Minkin S (2007). Mammographic density and the risk and detection of breast cancer. N Engl J Med.

[3] McCormack VA, dos Santos Silva I (2006). Breast density and parenchymal patterns as markers of breast cancer risk: a meta-analysis. Cancer Epidemiol Biomarkers Prev.

[4] Eriksson L, Czene K, Rosenberg L, Humphreys K, Hall P (2013). Possible influence of mammographic density on local and locoregional recurrence of breast cancer. Breast Cancer Res.

[5] Park CC, Rembert J, Chew K, Moore D, Kerlikowske K (2009). High mammographic breast density is independent predictor of local but not distant recurrence after lumpectomy and radiotherapy for invasive breast cancer. Int J Radiat Oncol Biol Phys.

[6] Cil T, Fishell E, Hanna W, Sun P, Rawlinson E, Narod SA, McCready DR (2009). Mammographic density and the risk of breast cancer recurrence after breast-conserving surgery. Cancer.

[7] Allred DC, Medina D (2008). The relevance of mouse models to understanding the development and progression of human breast cancer. J Mammary Gland Biol Neoplasia.

[8] Alowami S, Troup S, Al-Haddad S, Kirkpatrick I, Watson PH (2003). Mammographic density is related to stroma and stromal proteoglycan expression. Breast Cancer Res.

[9] Hawes D, Downey S, Pearce CL, Bartow S, Wan P, Pike MC, Wu AH (2006). Dense breast stromal tissue shows greatly increased concentration of breast epithelium but no increase in its proliferative activity. Breast Cancer Res.

[10] Lahlou H, Muller WJ (2011). Beta1-integrins signaling and mammary tumor progression in transgenic mouse models: implications for human breast cancer. Breast Cancer Res.

[11] van Dongen JA, Voogd AC, Fentiman IS, Legrand C, Sylvester RJ, Tong D, van der Schueren E, Helle PA, van Zijl K, Bartelink H (2000). Long-term results of a randomized trial comparing breast-conserving therapy with mastectomy: European Organization for Research and Treatment of Cancer 10801 trial. J Natl Cancer Inst.

[12] Poggi MM, Danforth DN, Sciuto LC, Smith SL, Steinberg SM, Liewehr DJ, Menard C, Lippman ME, Lichter AS, Altemus RM (2003). Eighteen-year results in the treatment of early breast carcinoma with mastectomy versus breast conservation therapy: the National Cancer Institute Randomized Trial. Cancer.

[13] Fisher B, Anderson S, Bryant J, Margolese RG, Deutsch M, Fisher ER, Jeong JH, Wolmark N (2002). Twenty-year follow-up of a randomized trial comparing total mastectomy, lumpectomy, and lumpectomy plus irradiation for the treatment of invasive breast cancer. N Engl J Med.

[14] Aapro MS, Forbes JF (2003). Three years' follow-up from the ATAC trial is sufficient to change clinical practice: a debate. Breast Cancer Res Treat.

[15] Yaffe MJ (2008). Mammographic density. Measurement of mammographic density. Breast Cancer Res.

[16] Wang J, Chang KJ, Kuo WH, Lee HT, Shih TT (2007). Efficacy of mammographic evaluation of breast cancer in women less than 40 years of age: experience from a single medical center in Taiwan. J Formos Med Assoc.

[17] Sovio U, Li J, Aitken Z, Humphreys K, Czene K, Moss S, Hall P, McCormack V, dos-Santos-Silva I (2014). Comparison of fully and semi-automated area-based methods for measuring mammographic density and predicting breast cancer risk. Br J Cancer.

[18] Sandberg ME, Li J, Hall P, Hartman M, Dos-Santos-Silva I, Humphreys K, Czene K (2013). Change of mammographic density predicts the risk of contralateral breast cancer - a case-control study. Breast Cancer Res.

[19] McGale P, Taylor C, Correa C, Cutter D, Duane F, Ewertz M, Gray R, Mannu G, Peto R, Whelan T (2014). Effect of radiotherapy after mastectomy and axillary surgery on 10-year recurrence and 20-year breast cancer mortality: meta-analysis of individual patient data for 8135 women in 22 randomised trials. Lancet.

[20] Gierach GL, Ichikawa L, Kerlikowske K, Brinton LA, Farhat GN, Vacek PM, Weaver DL, Schairer C, Taplin SH, Sherman ME (2012). Relationship between mammographic density and breast cancer death in the Breast Cancer Surveillance Consortium. J Natl Cancer Inst.

[21] Lin CH, Liau JY, Lu YS, Huang CS, Lee WC, Kuo KT, Shen YC, Kuo SH, Lan C, Liu JM (2009). Molecular subtypes of breast cancer emerging in young women in Taiwan: evidence for more than just westernization as a reason for the disease in Asia. Cancer Epidemiol Biomarkers Prev.

[22] Yao ES, Zhang H, Chen YY, Lee B, Chew K, Moore D, Park C (2007). Increased beta1 integrin is associated with decreased survival in invasive breast cancer. Cancer Res.

[23] dos Santos PB, Zanetti JS, Ribeiro-Silva A, Beltrao EI (2012). Beta 1 integrin predicts survival in breast cancer: a clinicopathological and immunohistochemical study. Diagn Pathol..

[24] Kim MY, Oskarsson T, Acharyya S, Nguyen DX, Zhang XH, Norton L, Massague J (2009). Tumor self-seeding by circulating cancer cells. Cell.

[25] Comen E, Norton L, Massague J (2011). Clinical implications of cancer self-seeding. Nat Rev Clin Oncol.

[26] Chen JL, Cheng JC, Kuo SH, Chan HM, Huang YS, Chen YH (2013). Prone breast forward intensity-modulated radiotherapy for Asian women with early left breast cancer: factors for cardiac sparing and clinical outcomes. J Radiat Res.

